# Revisiting the antidepressant-like effects of desipramine in male and female adult rats: sex disparities in neurochemical correlates

**DOI:** 10.1007/s43440-022-00372-1

**Published:** 2022-06-02

**Authors:** Sandra Ledesma-Corvi, M. Julia García-Fuster

**Affiliations:** 1 IUNICS, University of the Balearic Islands, Cra. de Valldemossa km 7.5, 07122 Palma, Spain; 2 Health Research Institute of the Balearic Islands (IdISBa), Palma, Spain

**Keywords:** Antidepressant, Rat, Hippocampus, Neuroplasticity, Sex

## Abstract

**Background:**

The preclinical antidepressant-like characterization of desipramine relied almost exclusively in male rodents, with only a few contradictory reports done in females. Given that most experiments assessed a single dose and/or timepoint of analysis after-treatment, this study evaluated potential sex-differences in the length of the antidepressant-like response induced by different doses of desipramine as well as the molecular underpinnings driving the different responses by sex.

**Methods:**

Male and female Sprague–Dawley rats were treated (i.p.) with 3 pulses of desipramine (5, 10 or 20 mg/kg) or vehicle (0.9% NaCl) within 24 h. The antidepressant-like effects were evaluated in the forced-swim test 1-h, 1- and 3-day post-treatment. The rate of cell proliferation and the regulation of key neuroplasticity markers (FADD, Cdk5, p35, p25) involved in antidepressant-like responses in the hippocampus were evaluated 1-h, 1-day and 5-day post-treatment.

**Results:**

Desipramine induced similar antidepressant-like effects in male and female rats (effective doses of 10 and 20 mg/kg, with effects that lasted up to 1-day post-treatment), without altering the rate of cell proliferation. However, some sex-differences emerged when evaluating neuroplasticity markers in the hippocampus, while no changes were observed for female rats, desipramine regulated FADD, Cdk-5 and p25 in males in a way that suggested neuroprotective actions.

**Conclusions:**

Our findings imply that while desipramine induced similar antidepressant-like responses for male and female rats, some differences emerged in the regulation of certain neuroplasticity markers, suggesting that distinctive molecular mechanisms might be participating in the therapeutic response of desipramine for both sexes.

## Introduction

There are well-known sex-differences in depression rates, with women being almost twice as likely to suffer depression as men (e.g., [1–3]). Moreover, an extensive body of research demonstrated sex differences in antidepressant efficacy (reviewed by [4, 5]), although the direction of the change is not completely clear; while generally speaking women seem less responsive and/or suffering more adverse effects than men, this appears especially true for tricyclic antidepressants; however, other reports suggested a better efficacy for females might also exist for drugs selectively inhibiting the serotonin reuptake (reviewed by [6]). These sex differences in efficacy might be explained by disparities in the pharmacokinetics and, to a lesser extent, pharmacodynamics effects of the drug (reviewed by [4–7]), which combined with the shortage of preclinical studies performed in female rats [8], justify the poor translation to the clinic, specially for female patients. Adjusting doses and/or timings, and/or even changing medications should be considered for each sex for an optimal clinical response.

In an effort to improve the scarce number of studies including sex as a biological variable [8–10], our research group (among several others) is centered in characterizing sex differences in antidepressant-like responses (e.g., [11–13]), including revisiting the antidepressant-like effects of desipramine in male and female rats which was the goal of the present study. Desipramine, a typical tricyclic antidepressant, is an effective and safe treatment prescribed for major depressive and other psychiatric disorders [14, 15]. Although, desipramine showed no significant sex differences for the treatment of multiple illnesses in children and adolescents [16], other prior pharmacological studies revealed an impact of sex in hormonal responses to desipramine in healthy young sibling pairs in a randomized cross-over experiment [17], as well as sex differences in pharmacokinetics (reviewed by [6]). The preclinical characterization of desipramine as an antidepressant relied almost exclusively in male naïve rodents (e.g., [18–20]) or male rodents exposed to stress [21], and generally utilizing the forced-swim test as a well-accepted experimental tool to screen drugs for its activity [22]. However, only a few preclinical studies included females, and showed contradictory results, with either signs of a significant antidepressant-like response [23] or a lack of efficacy [24]. In the context of these inconsistent effects, and given that most studies only assessed a single dose and/or timepoint of analysis after treatment, the present study evaluated potential sex differences in the length of the antidepressant-like response (1 h, 1 day and up to 5-day post-treatment) induced by different doses of desipramine (5, 10 and 20 mg/kg) in rats exposed to the forced-swim test, a model with strong predictive and discriminative validity, in which to further characterize the molecular underpinnings driving the potential different responses of desipramine by sex.

In particular, neural adaptations taking place in the hippocampus (reviewed by [25], and more recently by [26]), such as neurogenesis, are modulated in depressive-like states (neurogenic theory of depression; [27–29]), by most antidepressants [28, 30]), desipramine in particular [21, 31–33], and its ablation reduced the effects of certain antidepressants [34, 35], suggesting an important role for this particular process in the antidepressant-like response of certain drugs. Moreover, besides neurogenesis, other hippocampal plasticity events [25, 26] induced by desipramine also deserve further characterization, in terms of their role in controlling cell fate (balance between cell death vs. survival/plasticity), and since these processes might alter the basal rates of cell genesis, and participate in mediating some of the behavioral responses observed. For example, since depression has been linked to certain neurotoxic events (aberrant apoptosis, [36]) in humans and animals (reviewed by [25]), the role of antidepressants as neuroprotective agents (mediating anti-apoptotic actions) generated continued interest. Particularly, different antidepressant drugs, including desipramine, decreased pro-apoptotic signals (e.g., [37]) and promoted anti-apoptotic functions through the regulation of Fas-associated protein with death domain (FADD), and interestingly FADD levels correlated with the antidepressant-like response exerted by desipramine [19]. Finally, cyclin dependent kinase-5 (Cdk5), which is limited to post-mitotic neurons [39] and is linked to hippocampal neurogenesis by playing an important role in neuronal migration and hippocampal cell survival [42], also regulates depression-like behavior [38], and depends of co-factors p35 and p25 [40, 41]. While hippocampal Cdk-5 activity was increased in animal models of stress, it was reduced by antidepressants through redistribution of p35 from the cell membrane to the cytoplasm [43], suggesting that the Cdk-5/p35–p25 complex might play an important role in regulating antidepressant actions. Against this background, and since most molecular studies previously reported were performed almost exclusively in male rodents, this study deepened in the molecular sex differences induced by desipramine in male and female rats and developing throughout time.

## Experimental procedures

### Animals

Adult Sprague–Dawley rats (*n* = 142; 90 males and 52 females) were bred at the University of the Balearic Islands and housed in groups of 2–4 in standard cages in controlled environmental conditions (22 ºC, 70% humidity, and 12-h light/dark cycle, lights on at 8:00 a.m.) with *ad libitum* access to a standard diet and tap water. Prior to the experimental procedures, rats were habituated to being handled by the experimenter (for at least 2 days). All animal procedures were performed during the light period, followed ARRIVE guidelines [44, 45] and Directive 2010/63/EU of the European Parliament and of the Council, and were previously approved by the Local Bioethical Committee (University of the Balearic Islands; CEEA 58/04/16 and CEEA 155/12/20) and the Regional Government (Conselleria Medi Ambient, Agricultura i Pesca, Direcció General Agricultura i Ramaderia, Govern de les Illes Balears; Exp.: 2016/08/AEXP and 2021/05/AEXP) as stated in the Spanish Royal Decree 53/2013. All efforts were made to minimize the number of rats used and their suffering. In this context, to avoid inducing extra-stress in female rats during the experimental procedure, and since only a minor influence on the baseline response for naturally cycling females was reported in the forced-swim test (reviewed by [46]), and the cyclicity of females was not part of our research question (see [10]), the specific stages of the estrous cycle were not examined.

### Pharmacological treatments

**Fig. 1:**
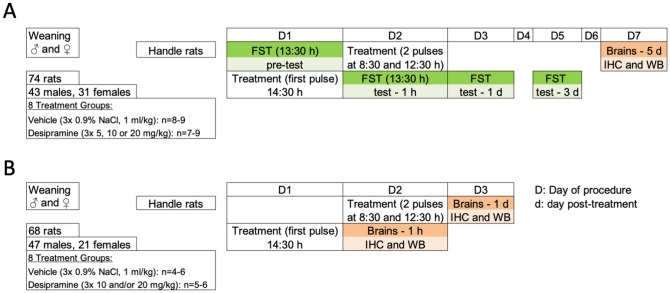
Experimental design. **A** Time-course of the antidepressant-like response induced by different doses of desipramine was evaluated in the forced-swim test (FST) 1-h and 1- and 3-day post-treatment in male and female rats (control-vehicle, *n* = 17 males and 8 females; desipramine 5 mg/kg, *n* = 9 males and 7 females; desipramine 10 mg/kg, *n* = 9 males and 8 females; desipramine 20 mg/kg; *n* = 8 males and 8 females). Prior to any drug treatment, rats were handled for some days and then exposed to a 15-min pre-forced-swim test (D1), followed by 3 pulses within a 24-h period of the corresponding treatment, and a sequence of 5-min forced-swim tests performed 1-h (D2), 1-day (D3) and 3-day (D5) post-treatment. Brains were collected 5-day post-treatment (D7) to evaluate potential neurochemical markers regulated by desipramine through immunohistochemistry (IHC) or western blot (WB) analyses. **B** Potential neurochemical markers regulated by desipramine were also evaluated by immunohistochemistry (IHC) or western blot (WB) analyses 1-h and 1-day post-treatment in male and female rats treated with the same drug regimens (control, 1 h: *n* = 12 males and 6 females; 1 day: *n* = 11 males and 4 females; desipramine 10 mg/kg, 1 h: *n* = 6 males; 1 day: *n* = 6 males; desipramine 20 mg/kg; 1 h: *n* = 6 males and 6 females; 1 day: *n* = 6 males and 5 females)

In a series of independent experiments (i.e., female and male rats were performed at different points in time), randomly allocated rats from each sex received (i.p.), 3 pulses (23, 5 and 1 h prior to behavioral or neurochemical testing; see Fig. 1) of desipramine (5, 10 or 20 mg/kg; doses selected from [19]) or saline (0.9% NaCl, 1 ml/kg, i.p.) within a period of 24 h, at the indicated times (see Fig. 1). The rats exposed to behavioral testing were sacrificed 5-day post-treatment (*n* = 74, see Fig. 1A), while other parallel experiments were performed in rats from each sex (with no behavioral testing) to collect brains at different timepoints after treatment (1-h and 1-day post-treatment; see Fig. 1B).

### Forced-swim test

A cohort of rats was used to evaluate the antidepressant-like potential of desipramine (control, *n* = 17 males and 8 females; desipramine 5 mg/kg, *n* = 9 males and 7 females; desipramine 10 mg/kg, *n* = 9 males and 8 females; desipramine 20 mg/kg; *n* = 8 males and 8 females; see Fig. 1A). To do so, we relied on the forced-swim test, with predictive validity for assessing antidepressant-like efficacy [22], and with prior reliable results from our research group (e.g., [19, 47, 48]), including the evaluation of the time-course length of the response (e.g., [11–13]). To perform this test, we used water tanks of 41 cm high × 32 cm diameter, filled with clean water for each rat to a depth of 25 cm and at a temperature of 25 ± 1 °C. Prior to any drug treatment, rats were exposed to a pre-test forced-swim session (15 min; D1, see Fig. 1A), followed by a sequence of 5-min tests performed 1-h, 1-day and 3-day post-treatment to evaluate duration of the antidepressant-like response of desipramine. Sessions were videotaped and analyzed by an experimenter blind to the treatment groups with Behavioral Tracker software (CA, USA) that provided a measure of the time rats spent immobile vs. active (i.e., climbing). Since experiments were done in different waves throughout the year, and given the described circannual changes in the duration of the immobility response of rats in the forced-swim test [49], among other factors also affecting baseline levels in the forced-swim test (reviewed by [50]), direct comparisons across studies (in terms of the individual seconds spent in each behavior) were not applicable and thus, results were calculated for each study as the percent change vs. the corresponding wave-control group for each sex.

### Tissue collection

Brain tissue from different cohort of rats was collected at the different timepoints of analysis (1 h, 1 and 5 days, see Fig. 1) in which to evaluate neuroplastic-like changes that might emerge during the course of treatment and that could explain the behavioral-like responses observed (control, 1 h: *n* = 12 males and 6 females; 1 day: *n* = 11 males and 4 females; desipramine 10 mg/kg, 1 h: *n* = 6 males; 1 day: *n* = 6 males; desipramine 20 mg/kg; 1 h: *n* = 6 males and 6 females; 1 day: *n* = 6 males and 5 females; see Fig. 1). All rats were killed by rapid decapitation and their brains removed at the indicated times. The left half-brain was quickly frozen in isopentane at – 30 ºC and stored at – 80 ºC, until it was cryostat-cut in 30 μm sections starting at around − 0.72 mm and up to -6.80 mm from Bregma, to cover the whole hippocampal extent (starting around − 1.72 from Bregma). Sections were slide-mounted and kept at – 80 ºC for the later analysis of the early stages of hippocampal neurogenesis (i.e., Ki-67 for recent cell proliferation) by immunohistochemistry. The right hippocampus was freshly dissected, frozen in liquid nitrogen, and kept at – 80 ºC until neuroplasticity markers (i.e., FADD, Cdk-5, p35-p25) were examined by Western blot experiments.

### Immunohistochemistry

The rate of cell proliferation was quantified in tissue sections (30 μm) that were post-fixed in 4% paraformaldehyde and exposed to several steps (i.e., antigen retrieval, blocking), including the incubation with the primary antibody anti-Ki-67 (1:40,000, kindly provided by Professors Huda Akil and Stanley J. Watson, University of Michigan, MI, USA) and as previously described (for further details see [51, 52]). Ki-67 + cells were quantified in the dentate gyrus with a Leica DMR light microscope (63 × objective lens) in every 8th section throughout the entire extent of the hippocampus following a modified unbiased stereological procedure [51, 52], and then the overall number of cells was multiplied by the sampling factor 8 to provide an estimate of the total number labeled cells per animal (e.g., [11, 51, 52]).

### Western blot

Following standardized protocols from our research group (e.g., [19, 53]), total brain proteins (40 μg) from rat hippocampal homogenates were resolved by electrophoresis on 10–12% SDS–PAGE minigels (Bio-Rad Laboratories, Hercules, CA, USA) and then transferred to nitrocellulose membranes that were incubated overnight at 4 ºC in blocking solution containing the appropriate primary antibodies (previously characterized, see [19, 53]). The vendors and conditions of the primary antibodies were the following: (1) Santa Cruz Biotechnology (CA, USA): anti-FADD (H-181) (dilution 1:5000) and anti-p35 (Ab-C19) (dilution 1:3000), (2) Lab Vision Corporation (CA, USA): anti-Cdk-5 (DC17) (dilution 1:1000), and (3) Sigma-Aldrich (MO, USA): anti-β-actin (AC-15) (dilution 1:10,000). Following the incubation (1 h) at room temperature with the corresponding secondary antibody (anti-rabbit or anti-mouse, 1:5000 dilution; Cell Signaling, MA, USA), target proteins were visualized by exposing membranes incubated with ECL reagents (Amersham, Buckinghamshire, UK) to an autoradiographic film (Amersham ECL Hyperfilm) for 1–60 min. The signal was quantified by densitometric scanning (GS-800 Imaging Calibrated Densitometer, Bio-Rad) and immunoreactivity of target proteins (i.e., FADD, Cdk-5, p35–p25 and β-actin) for each rat was calculated in each gel as the percent (%) change vs. the corresponding control group (1 or 5 days). Each sample was loaded at least 3 times in different gels and the mean value was used as a final estimate. β-actin served as a loading control, since its content was not altered by any treatment conditions (data not shown).

### Data analysis and data availability statement

Data were analyzed with GraphPad Prism, Version 9.3.1 (CA, USA). Bar graphs represent mean values ± standard error of the mean (SEM), and contain individual symbols for each rat, in line with the guidelines for displaying data and statistical methods in experimental pharmacology [54, 55]. Normal distribution was evaluated with Shapiro–Wilk normality test and thus parametric tests were used for data evaluation. Behavioral changes in the forced-swim test were evaluated by two-way repeated measures (RM) ANOVAs followed by Dunnett’s multiple comparisons tests when appropriate, in which Treatment (control vs. desipramine: 5, 10 and 20 mg/kg) and Time (1-h, 1- and 3-day post-treatment) were used as independent variables. The regulation of hippocampal cell genesis (Ki-67 + cells) and neuroplasticity markers were evaluated at each timepoint of analysis for male rats by one-way ANOVAs followed by Dunnett’s multiple comparison tests when appropriate, and for female rats by Student's *t* tests (control vs. 20 mg/kg groups). The variable Time was not included in the neurochemical analysis, since brains were collected at each timepoint of analysis from independent experiments (see Fig. 1), and given the potential basal differences in the regulation of certain molecular markers with the particular conditions at the time of sample collecting. The level of significance was set at *p* ≤ 0.05. The data that supports the findings of this study will be available upon reasonable request to the corresponding author.

## Results

### Desipramine induced similar antidepressant-like responses in male and female rats

**Fig. 2:**
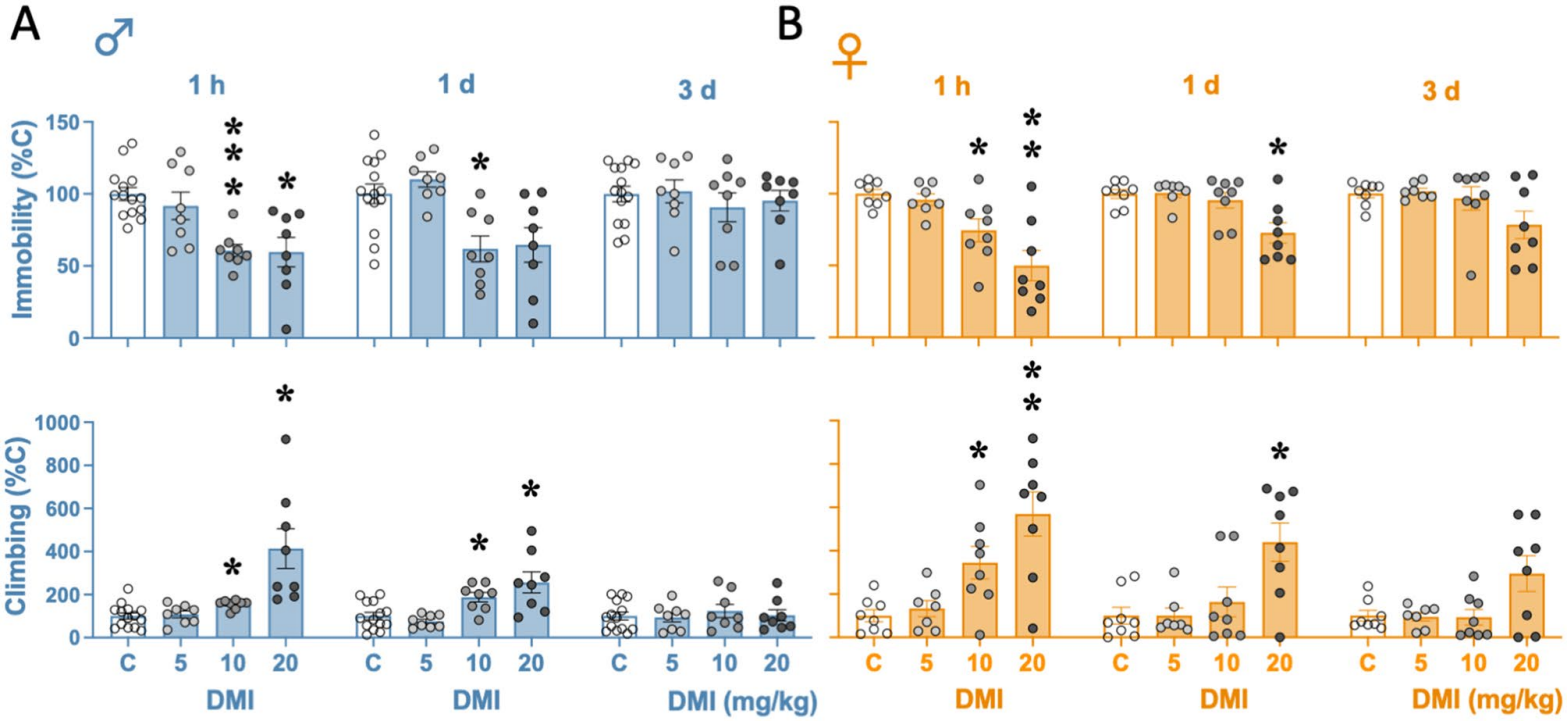
Evaluating the time-course of the antidepressant-like response of different doses of desipramine in male and female rats. Groups of treatment (i.p., 3 pulses within a 24-h period): control (C: 0.9% NaCl, 1 ml/kg; *n* = 17 males and 8 females), desipramine (DMI) 5 mg/kg (*n* = 9 males and 7 females), 10 mg/kg (*n* = 9 males and 8 females), or 20 mg/kg (*n* = 8 males and 8 females). Columns represent mean ± SEM of the % time spent immobile or climbing (individual symbols are shown for each rat). Two-way RM ANOVAs followed by Dunnett's multiple comparisons tests: ***p < 0.001, **p < 0.01 or *p < 0.05 when compared to control rats at the indicated timepoint of analysis

The antidepressant-like effect of desipramine was evaluated across time (1-h, 1- and 3-day post-treatment) in the forced-swim test in male rats by two-way RM ANOVAs. The results showed significant Treatment x Day interactions for immobility (*F*_6,68_ = 4.82, *p* < 0.001) and climbing (*F*_6,68_ = 10.78, *p* < 0.001; Fig. 2A). Dunnett’s *post-hoc* comparisons revealed that desipramine decreased immobility in a dose-dependent manner as observed 1-h (5 mg/kg: -8 ± 10%, *p* = 0.799, n.s.; 10 mg/kg: − 39 ± 6%, ***p < 0.001; 20 mg/kg: − 40 ± 11%, *p = 0.013) and 1-day post-treatment (5 mg/kg: + 10 ± 8%, *p* = 0.554, n.s.; 10 mg/kg: − 38 ± 11%, *p = 0.011; 20 mg/kg: − 35 ± 14%, *p* = 0.062, n.s.) and as compared with control-treated rats. Similarly, Dunnett’s *post-hoc* comparisons for climbing showed significant increases 1-h (5 mg/kg: + 8 ± 21%, *p* = 0.971, n.s.; 10 mg/kg: + 55 ± 16%, *p = 0.010; 20 mg/kg: − 314 ± 94%, *p = 0.030) and 1-day post-treatment (5 mg/kg: − 25 ± 20%, *p* = 0.496, n.s.; 10 mg/kg: + 88 ± 28%, *p = 0.017; 20 mg/kg: + 157 ± 52%, *p = 0.039) and as compared with control-treated rats (Fig. 2A). For female rats, similar results were observed: Treatment x Day interactions for immobility (*F*_6,54_ = 3.49, *p* = 0.006) and climbing (*F*_6,54_ = 4.32, *p* = 0.001; Fig. 2B) and significant Dunnett’s *post-hoc* comparisons for immobility (1 h: 5 mg/kg, − 4 ± 5%, *p* = 0.756, n.s.; 10 mg/kg, -26 ± 9%, *p = 0.037; 20 mg/kg, − 50 ± 11%, **p = 0.005; 1 day: 5 mg/kg, + 1 ± 4%, *p* = 0.997, n.s.; 10 mg/kg, − 4 ± 6%, *p* = 0.830, n.s.; 20 mg/kg, − 27 ± 8%, *p = 0.015) and climbing (1 h: 5 mg/kg, + 33 ± 47%, *p* = 0.820, n.s.; 10 mg/kg, + 244 ± 80%, *p = 0.035; 20 mg/kg, + 470 ± 106%, **p = 0.005; 1 day: 5 mg/kg, 0 ± 53%, *p* > 0.999, n.s.; 10 mg/kg, + 64 ± 79%, *p* = 0.767, n.s.; 20 mg/kg, + 339 ± 97%, *p = 0.015). All the changes induced by desipramine on immobility and climbing in male and female rats returned to normal 3-day post-treatment (see Fig. 2A and 2B).

### Desipramine did not regulate the rate of cell proliferation in the dentate gyrus of male and female rats

**Fig. 3:**
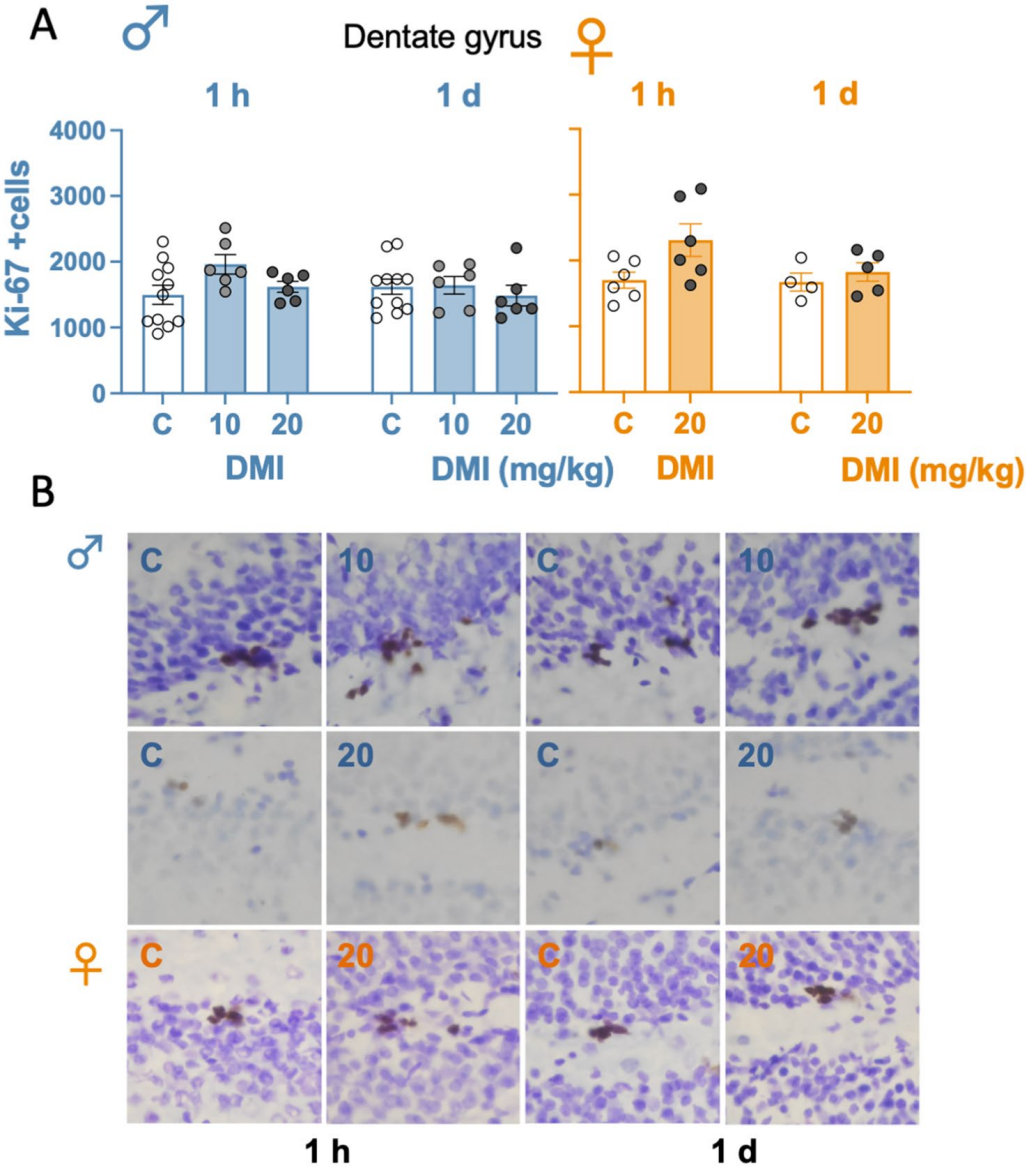
Evaluating the effects of desipramine on the rate of hippocampal cell proliferation in male and female rats as measured 1-h and 1-day post-treatment by immunohistochemistry. **A** Quantitative analysis of Ki-67 + cells in the left dentate gyrus, performed in a light microscope with a 63 × lens. Groups of treatment (i.p., 3 pulses within a 24-h period): control (C) (0.9% NaCl, 1 ml/kg: 1 h, *n* = 12 males and 6 females; 1 day, *n* = 11 males and 4 females), desipramine (DMI) 10 mg/kg (1 h: *n* = 6 males; 1 day: *n* = 6 males) or 20 mg/kg (1 h: *n* = 6 males and 6 females; 1 day: *n* = 6 males and 5 females). Columns represent mean ± SEM of Ki-67 + cells (individual symbols are shown for each rat). One-way ANOVAs did not detect any significant changes. **B** Representative images showing individual Ki-67 + cells (brown labeling in the blue granular layer) taken with a light microscope using a 40 × objective lens to identify individual cells

The effect of desipramine on regulating an early marker of hippocampal neurogenesis (i.e., cell proliferation by labeling Ki-67 + cells) was evaluated 1-h and 1-day post-treatment (timepoints with an observed antidepressant-like effect). The results showed that desipramine did not significantly alter the number of Ki-67 + cells observed in the hippocampus of male (1 h: *F*_2,20_ = 2.67, *p* = 0.094; 1 day: *F*_2,20_ = 0.34, *p* = 0.715) and female (1 h: *t* = 2.21, *df* = 10, *p* = 0.052; 1 day: *t* = 0.77, *df* = 7, *p* = 0.467) rats (see Fig. 3).

### Desipramine modulated certain neuroplasticity markers in the hippocampus of male but not female rats

**Fig. 4:**
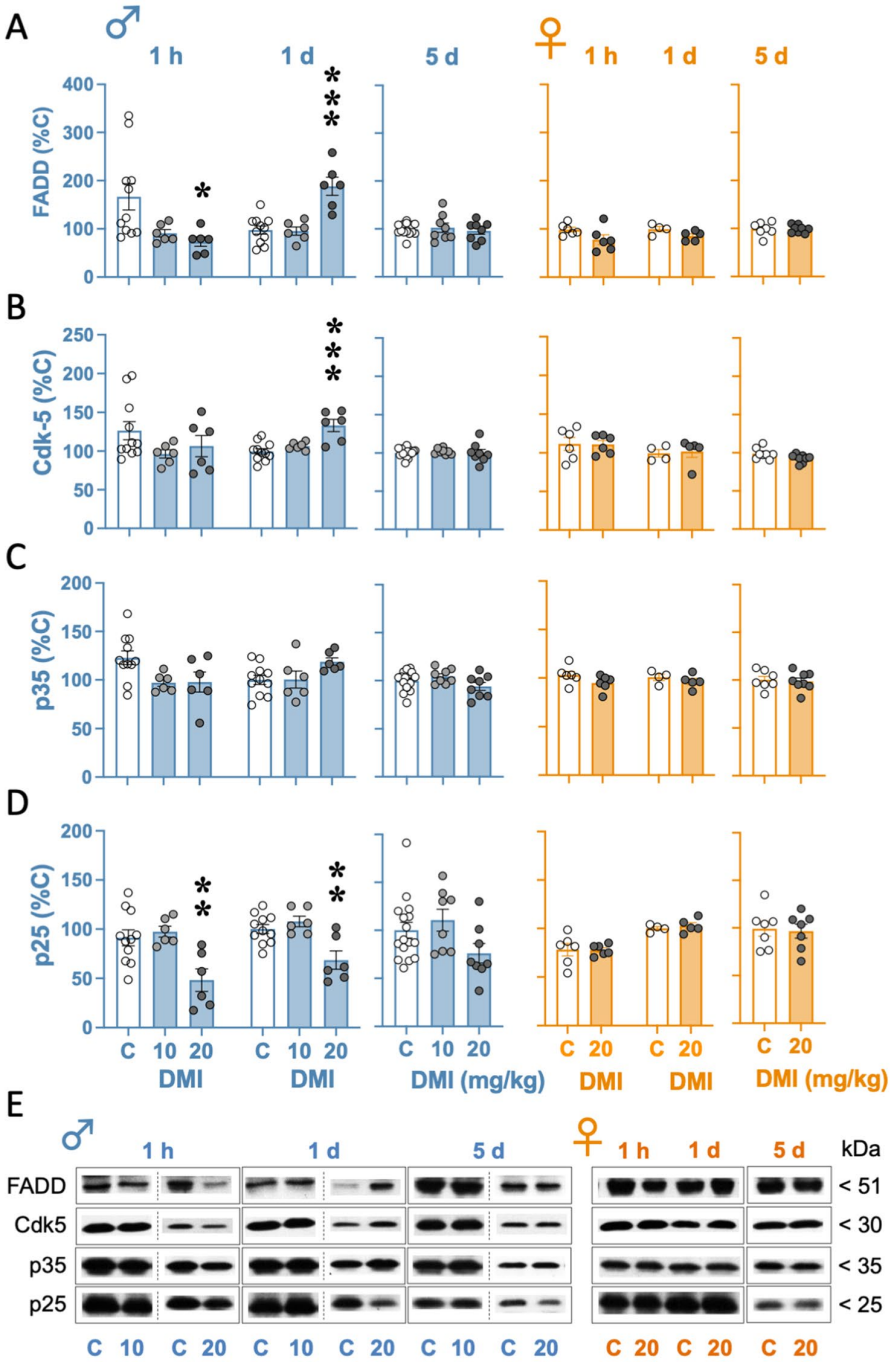
Evaluating the effects of desipramine on hippocampal neuroplasticity markers in male and female rats as measured 1-h and 1- and 5-day post-treatment by western blot. **A** FADD, **B** Cdk5, **C** p35 and **D** p25 protein content in hippocampal samples. Groups of treatment (i.p., 3 pulses within a 24-h period): control (C) (0.9% NaCl, 1 ml/kg; 1 h: *n* = 12 males and 6 females; 1 day: *n* = 11 males and 4 females), desipramine (DMI) 10 mg/kg (1 h: *n* = 6 males; 1 day: *n* = 6 males) or 20 mg/kg (1 h: *n* = 6 males and 6 females; 1 day: *n* = 6 males and 5 females). Columns represent mean ± SEM of *n* experiments per group and expressed as % control (individual values are shown for each rat in symbols). One-way ANOVAs at each timepoint of analysis followed by Dunnett's multiple comparisons tests when appropriate: *p < 0.05, **p < 0.01 and *p < 0.05 vs. control rats at the indicated timepoint of analysis. **E** Representative immunoblots depicting labeling of target proteins at each timepoint of analysis

When assessing FADD hippocampal regulation, one-way ANOVAs detected significant effects 1 h (*F*_2,20_ = 4.81, *p* = 0.020) and 1 day (*F*_2,20_ = 18.11, *p* < 0.001), but not 5 days (*F*_2,29_ = 0.25, *p* = 0.779) post-treatment (Fig. 4A). Dunnett’s multiple comparisons tests revealed that the dose of 20 mg/kg of desipramine decreased FADD content 1-h post-treatment (− 93 ± 33%, *p = 0.022), while increased it 1-day post-treatment (+ 90 ± 16%, ***p < 0.001) as compared to their respective control treated rats (Fig. 4A). No significant effects were observed for female rats (1 h: *t* = 1.86, *df* = 10, *p* = 0.093; 1 day: *t* = 2.03, *df* = 7, *p* = 0.082; 5 days: *t* = 0.06, *df* = 13, *p* = 0.956; Fig. 4A).

As for Cdk5, one-way ANOVAs detected significant effects 1-day (*F*_2,20_ = 12.72, *p* < 0.001), but not 1-h (*F*_2,20_ = 1.82, *p* = 0.189) or 5-day (*F*_2,29_ = 0.17, *p* = 0.848) post-treatment (Fig. 4B). Dunnett’s multiple comparisons tests revealed that the dose of 20 mg/kg of desipramine increased Cdk5 protein content 1-day post-treatment (+ 33 ± 7%, *p = 0.022) as compared to their respective control treated rats (Fig. 4B). No significant effects were observed for female rats (1 h: *t* = 0.07, *df* = 10, *p* = 0.948; 1 day: *t* = 0.25, *df* = 7, *p* = 0.812; 5 days: *t* = 2.06, *df* = 14, *p* = 0.059; Fig. 4B).

The analysis of hippocampal p35 revealed a significant effect 1 h (*F*_2,20_ = 4.05, *p* = 0.033), but not 1-day (*F*_2,20_ = 2.92, *p* = 0.077) or 5-day (*F*_2,29_ = 2.24, *p* = 0.125) post-treatment (Fig. 4C). However, no significant pairwise comparisons were revealed by Dunnett's test 1-h post-treatment. Similarly, to the regulation of the other proteins, no significant effects were observed for female rats (1 h: *t* = 1.66, *df* = 10, *p* = 0.129; 1 day: *t* = 0.94, *df* = 7, *p* = 0.380; 5 days: *t* = 0.31, *df* = 13, *p* = 0.759; Fig. 4C).

Finally, when assessing p25 hippocampal regulation, one-way ANOVAs detected significant effects 1 h (*F*_2,20_ = 7.70, *p* = 0.003) and 1 day (*F*_2,20_ = 9.47, *p* = 0.001), but not 5-day (*F*_2,29_ = 2.52, *p* = 0.098) post-treatment (Fig. 4D). Dunnett’s multiple comparisons tests revealed that the dose of 20 mg/kg of desipramine decreased p25 content as measured 1-h (− 43 ± 12%, **p = 0.005) and 1-day (− 31 ± 9%, **p = 0.003) post-treatment, and as compared to their respective control groups (Fig. 4C). Again, no significant effects were observed for female rats (1 h: *t* = 0.04, *df* = 10, *p* = 0.968; 1 day: *t* = 0.65, *df* = 7, *p* = 0.534; 5 days: *t* = 0.24, *df* = 12, *p* = 0.812; Fig. 4D).

## Discussion

This study compared the course of the antidepressant-like potential of different doses of desipramine administration and deepened in the putative sex differences in neuroplasticity signaling partners modulated in the hippocampus, a key region for affect regulation. The main results showed that desipramine induced antidepressant-like effects of similar magnitude and duration for both sexes (effective doses of 10 and 20 mg/kg, with responses that lasted up to 1-day post-treatment), without altering the rate of cell proliferation. However, desipramine regulated some neuroplasticity markers exclusively in male rats (FADD, Cdk5 and p25), in a way that suggested neuroprotective actions, proposing that distinctive molecular mechanisms might be participating in the therapeutic response of desipramine for both sexes.

The antidepressant-like response of desipramine was observed with a similar magnitude in male and female rats, as decreased rates of immobility and increased climbing in the forced-swim test, therefore, presenting preclinical evidence of efficacy for both sexes. The effective doses were 10 and 20 mg/kg (3 pulses within 24 h) and efficacy was observed 1 h and up to 1-day post-treatment. Our data extended the published literature in the field by showing the time-course of the antidepressant-like response, since most previous studies only evaluated a single timepoint (mostly 1-h post-treatment) while also including efficacious doses for female rats. In particular, desipramine induced a potent antidepressant-like response as early as 1-h post-treatment, which was still present with a similar magnitude 1 day later, but dissipated 3-day post-treatment. Some prior studies suggested that lower doses of desipramine were ineffective when administered acutely (2 doses of 1, 2 or 5 mg/kg over 24-h, test performed 1-h post-injection), but induced an antidepressant-like effect only when given chronically (5 mg/kg, 15 injections [18]) in male Sprague–Dawley rats. However, other acute studies with a higher dose range (5, 10 and 20 mg/kg × 3 doses in 24 h), similar to the one evaluated here, also demonstrated diminished immobility in the forced-swim test in male rats [56] (see [19] for our own prior results with 3 × 20 mg/kg). As for female rats, only a few preclinical studies included females; while one demonstrated a significant antidepressant-like response with a 3 × 10 mg/kg dose in ovariectomized rats [23], the other one showed no effects for female Sprague–Dawley rats, even though it tested doses up to 3 × 10 mg/kg, which were effective in the present study [24]. The authors of that study discussed that their experimental groups had a limited sample size that might have prevented observing subtle differences (see [24]). Interestingly, as expected for antidepressants that enhance norepinephrine neurotransmission [18, 56, 57], desipramine increased climbing in the forced-swim test for male rats, but interestingly, the same pattern was also observed for female rats. Therefore, contrary to other treatments that had clearly shown sex differences in antidepressant-like responses, with a drop in efficacy and/or even an inefficacious response for females (i.e., electroconvulsive therapy, see [11]; or more novel compounds, such as ketamine and cannabidiol, see [12, 13]), desipramine has proven a similar behavioral response in male and female rats.

In terms of the potential molecular mechanisms mediating the therapeutic response, the study evaluated an early stage of hippocampal neurogenesis (cell proliferation, Ki-67 + cells), but showed no changes for either sex. These negative effects were consistent with prior findings suggesting the need for a repeated drug treatment (14 days) to promote hippocampal cell proliferation [21, 32]. So far, the results suggested that while acute desipramine is sufficient to induce an antidepressant-like response of similar magnitude for males and female rats, longer treatments might be needed to increase cell proliferation, suggesting this mechanism could not fully mediate and/or explain the behavioral outcome. In this line of thought, previous studies with other antidepressants, both from our group [47] and others [58], showed a dissociation between emotional behavior and neurogenesis regulation, suggesting that these responses might occur independently or, at least, at different time-courses and/or lengths of treatment. These results contradict prior studies suggesting the need of a neurogenesis activation for the antidepressant-like response to occur [34], or for the observed parallel regulation of both effects [59–61], indicating that other factors/molecular players might participate in the observed antidepressant-like response induced by desipramine.

In this context, we evaluated the regulation of several key hippocampal cell-fate markers. Overall, the present results complemented prior data from our group showing that acute desipramine induced neuroprotective actions by decreasing FADD 1-h post-treatment in rat brain cortex [19], to include another brain region (hippocampus) regulated in a similar fashion, as well as the course of FADD regulation (up to 5-day post-treatment), and the lack of effects observed in female rats. Following the initial decrease in FADD 1-h post-treatment, desipramine increased FADD content 1-day post-treatment (i.e., rebound effect after the acute initiation of an anti-apoptotic response, see prior similar results by [62]) and all levels were normalized 5-day post-treatment, suggesting a role for this neuroplasticity marker in the effects induced by desipramine. Moreover, this is the first study evaluating FADD hippocampal regulation by desipramine in female rats, proving sex disparities in its regulation that deserve further characterization.

As for the rest of the hippocampal markers evaluated (Cdk5, p35 and p25), some indications of neuroprotective actions were also observed as measured 1-h post-treatment exclusively in male rats, such as the decrease in neurotoxic p25 marker. Interestingly, these effects were also present 1-day post-treatment, together with an increase in hippocampal Cdk5. These effects normalized 5-day post-treatment, when the behavioral response dissipated. In this context, the activation of the apoptotic-pathway through FasL, in which FADD is the intracellular adaptor to Fas receptor, increased the transcription of p35 through ERK_1/2_ pathway [63], thus suggesting an indirect connection for FADD and Cdk5, p35–p25 signaling molecules. In addition, the regional inhibition of Cdk-5 in the dentate gyrus blocked depressive-like behavior (induced by chronic mild stress) in rats, whereas the overexpression of p35 blocked the antidepressant-like effect induced by venlafaxine [43], suggesting a key role for the complex Cdk5/p35–p25 in the regulation of antidepressant-like responses. Moreover, Cdk-5 activity was not required for the proliferation or differentiation of neuronal stem cells, but was essential for the survival, migration and maturation of newborn granule cells [64]. Overall, the molecular results suggested the involvement of FADD, Cdk5 and p35–p25 complex in the neuroplastic actions taking place in the hippocampus [26] and mediated by desipramine but exclusively in male rats, demonstrating that although the behavioral effects were similar, clear sex differences were observed in the molecular events driving those responses, that deserve further study.

Taken together, these results showed that while acute desipramine was capable of inducing antidepressant-like effects of the same magnitude and duration in male and female rats, the regulation of some neuroplasticity markers, that paralleled the behavioral response, was only detected in male rats. Overall, the results suggested that the regulation of FADD, Cdk-5 and p35–p25 proteins could be common markers mediating some of the early mechanisms taking place right after drug exposure and up to 1-day post-treatment while matching the course of the observed behavioral response. On the contrary, the regulation of an early stage of adult hippocampal neurogenesis, such as cell proliferation, might require a prolonged treatment exposure to desipramine, since no effects were observed following this acute paradigm. Overall, these molecular correlates were exclusively regulated in male rats, suggesting other markers might be participating in the behavioral response observed in females. Further studies are required to complement these results and deepen into the neurochemical differences driven by sex in the response mediated by desipramine, since these disparities might be responsible for the poor clinical translation occasionally described.
